# Hyperthymic traits, major depression and bipolar spectrum, review and case report

**DOI:** 10.1192/j.eurpsy.2021.1651

**Published:** 2021-08-13

**Authors:** M. García Moreno, A. De Cós Milas, L. Beatobe Carreño, P. Del Sol Calderon, Á. Izquierdo De La Puente

**Affiliations:** 1 Psychiatry, HOSPITAL UNIVERSITARIO PUERTA DE HIERRO MAJADAHONDA, MADRID, Spain; 2 Psychiatry, HOSPITAL UNIVERSITARIO DE MÓSTOLES, MADRID, Spain

**Keywords:** bipolar disorder, bipolar spectrum, hyperthymic temperament, Major Depression

## Abstract

**Introduction:**

Akiskal proposed the bipolar spectrum concept with the aim of including those patients with atypical depressive presentations and mood temperaments. Also Koukopoulos accepted this proposal in those patients with poor response to antidepressants or highly recurrent course. Concretely bipolar disorder type IV was defined as clinical depression based on a lifelong hyperthymic temperament. Some years after DSM-III several experts in bipolar disorder continued in this work line even though DSM-IV and most recent DSM-V not considered to include this concept as a new diagnostic category.

**Objectives:**

To present a theoretical and practical review about bipolar spectrum and its relationship with hyperthymic traits.

**Methods:**

We carry out a literature review about bipolar spectrum, accompanied by the clinical description of one patient with major depressive disorder and hyperthymic traits base.

**Results:**

45 years old female referred to our outpatient mental health service after episode of voluntary drug overdose. She presented long evolution depressive symptoms (sadness, apathy, anhedonia, anergy, irritability, anxiety, emotional lability, early awakening, social withdrawal, self-care neglect, hopelessness, cognitive failures, guilt feelings and death ideas) with onset in postpartum. She reported a previous depressive episode 9 years ago with good response to fluoxetine. Hyperthimic traits were described but no history of manic symptoms. An erratic evolution was observed with various antidepressant treatment and finally improved adding mood stabilizer.

**Conclusions:**

We must propose to consider the diagnosis of bipolar spectrum in order to treat effectively patients with major depression dissorder and hyperthymic temperament in absence of manic symptoms.

**Disclosure:**

No significant relationships.

